# Mapping of Apoptin-interaction with BCR-ABL1, and development of apoptin-based targeted therapy

**DOI:** 10.18632/oncotarget.2278

**Published:** 2014-08-13

**Authors:** Jaganmohan R. Jangamreddy, Soumya Panigrahi, Kourosh Lotfi, Manisha Yadav, Subbareddy Maddika, Anil Kumar Tripathi, Sabyasachi Sanyal, Marek J. Łos

**Affiliations:** ^1^ Dept. Clinical & Experimental Medicine, Integrative Regenerative Med. Center (IGEN), Linköping University, Sweden; ^2^ Dept. Medicine/ Infectious Diseases, Case Western Reserve University, Cleveland, OH 44106, USA; ^3^ Dept. of Medical and Health Sciences, Linköping University, Department of Hematology, County Council of Östergötland, Linköping, Sweden; ^4^ Division of Biochemistry, CSIR-Central Drug Research Institute, 10, Janakipuram Extn, Sitapur Rd, Lucknow 226031, UP, India; ^5^ Laboratory of Cell Death & Survival, Centre for DNA Fingerprinting and Diagnostics (CDFD), Hyderabad, India; ^6^ Department of Clinical Hematology and Medical Oncology, King George's Medical University, Lucknow 226003, Uttar Pradesh, India; ^7^ Department of Pathology, Pomeranian Medical University, Szczecin, Poland

**Keywords:** apoptin, BCR-ABL1, CML, imatinib, STAT5

## Abstract

Majority of CHRONIC myeloid leukemia patients experience an adequate therapeutic effect from imatinib however, 26–37% of patients discontinue imatinib therapy due to a suboptimal response or intolerance. Here we investigated derivatives of apoptin, a chicken anemia viral protein with selective toxicity towards cancer cells, which can be directed towards inhibiting multiple hyperactive kinases including BCR-ABL1. Our earlier studies revealed that a proline-rich segment of apoptin interacts with the SH3 domain of fusion protein BCR-ABL1 (p210) and acts as a negative regulator of BCR-ABL1 kinase and its downstream targets. In this study we show for the first time, the therapeutic potential of apoptin-derived decapeptide for the treatment of CML by establishing the minimal region of apoptin interaction domain with BCR-ABL1. We further show that the apoptin decapeptide is able to inhibit BCR-ABL1 down stream target c-Myc with a comparable efficacy to full-length apoptin and Imatinib. The synthetic apoptin is able to inhibit cell proliferation in murine (32D^p210^), human cell line (K562), and *ex vivo* in both imatinib-resistant and imatinib sensitive CML patient samples. The apoptin based single or combination therapy may be an additional option in CML treatment and eventually be feasible as curative therapy.

## INTRODUCTION

The identification of BCR-ABL1 as the single cause of CML, enabled novel therapeutic approaches aiming at the inhibition of the tyrosine kinase activity of BCR-ABL1 [1]. BCR-ABL1 is formed (Philadelphia (Ph) chromosome) as a reciprocal translocation between the long arms of chromosomes 9 and 22 resulting in the *breakpoint cluster region* (*BCR*) gene from chromosome 9 being positioned next to the *c-abl oncogene 1* (*ABL1*) in chromosome 22. The leukemogenic properties of BCR-ABL1 originate from the constitutive tyrosine kinase activity of the ABL1-encoded part of the protein in combination with a region in the BCR moiety that facilitates dimerization of BCR-ABL1. Dimerized BCR-ABL1 autophosphorylates itself at tyrosine residues that promote the recruitment and activation of the intracellular signaling protein complex of growth factor receptor-bound protein 2 (GRB2), GRB2-associated binding protein 2 (GAB2) and son-of-sevenless (SOS). The activated GRB2/GAB2/SOS complex in turn activates several downstream signaling cascades including the RAS/mitogen-activated protein kinase (MAPK), signal transducer and activator of transcription 5 (STAT5) and phosphatidylinositol 3-kinase (PI3K)/AKT pathways [2, 3]. Collectively, these pathways influence genetic transcription so that uncontrolled cell survival, proliferation, and anti-apoptotic pathways are promoted and the CML cell clone has been created.

The first tyrosine kinase inhibitor (TKI) imatinib binds to and block the ATP-binding pocket of the BCR-ABL1 protein, thereby preventing the essential access of ATP for tyrosine kinase activity [4, 5]. Consequently, imatinib shuts down all downstream signaling events from BCR-ABL1 and specifically inhibits survival of CML cells. Although imatinib is a superior therapeutic alternative compared to all previously investigated options, there is still room for improvements as 20-30% of patients develop resistance to imatinib [6] and 7-9% of patients progress into accelerated phase (AP) or blast crises (BC) on imatinib treatment during five years of first-line treatment [7, 8]. Furthermore, 10% of patients discontinue imatinib therapy due to adverse effects [9]. The second generation TKIs (2GTKIs) induce deeper and faster molecular and cytogenetic responses than imatinib. In spite of this, there is no clear improvement in overall survival. Therefore, targeting multiple signaling pathways is necessary for an improved therapeutic outcome [10].

Apoptin is a 14 KD viral protein (chicken anemia virus protein-3, VP3) and known to induce apoptosis with selective toxicity towards cancer cells. Apoptin mediated cell toxicity is dependent on its cellular localization, as nuclear localization promote cell death while cytoplasmic presence does not render such cell death [11, 12]. Studies from our lab and several others show the p53-independent, mitochondrial mediated caspase-3 dependent cell death by apoptin among cancer cells [13-15]. Several apoptin interacting molecules including Nur77, BCL-family, Anaphase promoting complex (APC) and PI3K signaling pathways are studied in the process to unravel the cancer cell specific toxicity of apoptin [13, 14, 16-18].

Our previous studies indicated that Apoptin interacts and inhibits Abl/BCR-ABL1 and its various downstream targets including STAT5, CrkL and c-Myc [19]. Here we describe the identification of apoptin and its derivatives as specific interacting elements with the SH3-domain of BCR-ABL1. We further map the region of apoptin responsible for its interaction with BCR-ABL1 using various deletion mutants of apoptin and confirm interaction of apoptin-derived constructs with Abl/ BCR-ABL1 by GST-apoptin pull-down assay. Furthermore, we designed a decapeptide spanning the apoptin-interaction domain, and tested its toxicity towards BCR-ABL1 positive and control cells. Inspired by the results on the established cell lines we tested the toxicity of apoptin derived decapeptide on the CML patient derived blood samples and show its effective function even among the imatinib resistant patient primary samples. Thus, with this study we provide apoptin-derived decapeptide as a better alternative therapeutic agent for the treatment of leukemia (or complement as combination therapy) as opposed to TKI to which cells often develop resistance. Since BCR-ABL1 is not expressed in normal cells apoptin decapeptide will be an ideal drug target as it leaves normal cells unaffected by acting as a selective BCR-ABL1 inhibitor.

## RESULTS

### Apoptin induces apoptosis in *BCR-ABL1* positive and immortalized cells

**Figure 1:**
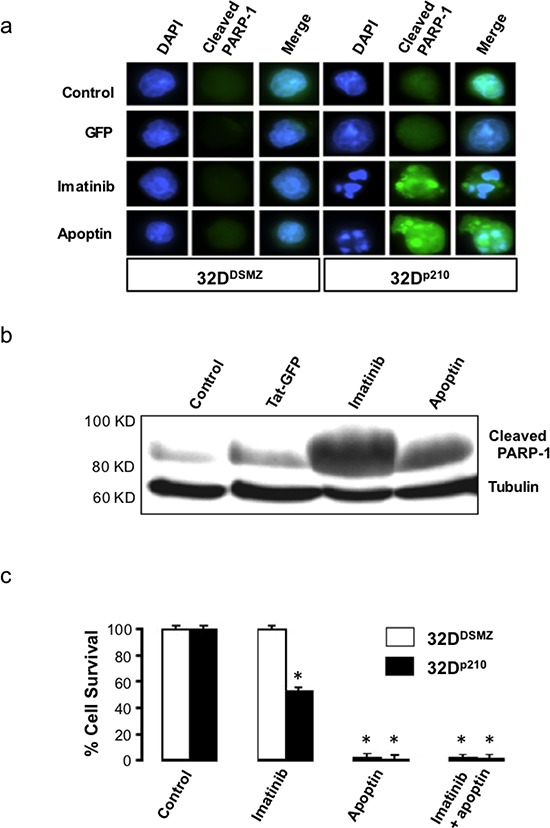
Apoptin kills both BCR-ABL1 positive and negative cells **(a)** Elevated level of cleaved PARP-1 in 32D^p210^ cells treated with apoptin. **(b)** Appearance of cleaved PARP-1 and induction of apoptosis in Bcr-Abl expressing 32D^p210^ cells when treated with apoptin or imatinib; **(c)** The effects of apoptin on the survival of Bcr-Abl expressing cells as determined by Nicoletti method. N=3. *P<0.03.

Apoptin triggers the activation of caspases via the intrinsic/mitochondrial death pathway, and not the death receptor/extrinsic pathway in cancer cells [15]. To further verify the nature of apoptin induced cell death among BCR-ABL1 expressing leukemia cells, we compared nuclear morphology of the apoptin/imatinib untreated and treated 32D^DSMZ^ and 32D^p210^ cells to study the features of apoptotic nuclei (Fig. 1a). Furthermore, we estimated the presence of cleaved PARP-1, which is a key target of activated caspase-3, or -7 in pro-apoptotic cells by Western blot analysis and immunocytochemistry (Fig. 1a and 1b). In these experiments, the characteristic apoptotic nuclear morphology and presence of cleaved PARP-1 in the cytoplasm of apoptin treated 32D^p210^ cells clearly indicate the induction of apoptosis following the application of apoptin (Fig. 1a and 1b).

To study the biological activity of the cell-penetrating Tat-apoptin on 32^p210^ cells expressing BCR-ABL1^p210^, we treated with Tat-apoptin (1μM) and cell survival was assessed by MTT assay at different time points. Treatment of 32^p210^ cell lines with either Tat-Apoptin or the positive control Imatinib caused significant cell death (p < 0.03) as compared to the negative control group receiving Tat-GFP treatment (Fig. 1c). This result further confirms the very nature of anti-proliferative effect of apoptin that does not rely on a single target, but it instead affects multiple cell growth pathways and thus the development of apoptin resistance is less likely.

### Apoptin interacts with the Src homology domain 3 of *BCR-ABL1*

**Figure 2:**
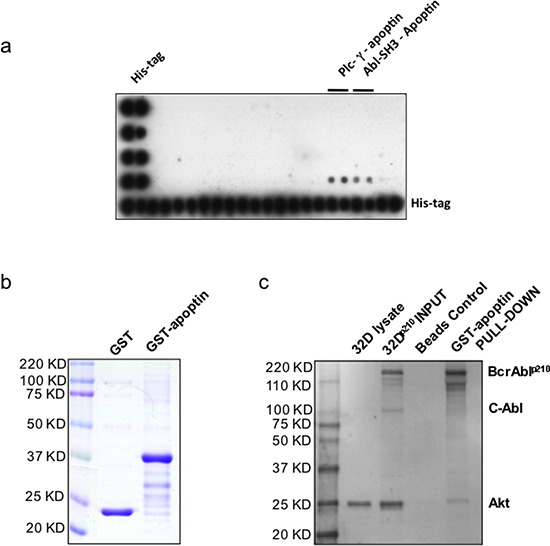
Apoptin interacts with the SH3-domain of Abl – confirmation of apoptin's interaction with BCR-ABL1 **(a)** TransSignal SH3 Domain Array1 interaction of apoptin and SH3 domains of Abl (D 3, 4); **(b)** Production of recombinant GST conjugated apoptin. **(c)** Akt, Apoptin & Bcr-Abl Interaction: Class lb SH3 ligand specifically ‘pulls down’ its corresponding protein-protein interaction domains.

To investigate possible interactions of apoptin with the SH3 domains of a series of proteins we first performed a protein array-based study. Several well-characterized SH3 domains were previously identified as the potential sites critical to ligand binding on the basis of alignment with their structures [20]. We performed a high stringency SH3 domain interaction array screening, which indicated that apoptin strongly interacts with the SH3 domain of some proteins including Abl (Fig. 2a). The above observation was further confirmed by GST-apoptin and BCR-ABL1^p210^ ‘pull-down assay’ using both BCR-ABL1-positive (32D^p210^), and -negative (32D^DSMZ^) cell lines (Fig. 2b, 2c) where full length BCR-ABL1^p210^ with intact SH3 domain showed interaction with GST-apoptin and were ‘pulled-down’ by glutathione sepharose beads. Interestingly when the same blot was incubated with a second primary antibody against Akt, a band positively correlating with the molecular weight of Akt was identified in the GST-apoptin BCR-ABL1 pull-down product indicating a co-reactivity (Fig. 2c).

### Specific interacting motif of apoptin is responsible for its interaction with *BCR-ABL1*

**Figure 3:**
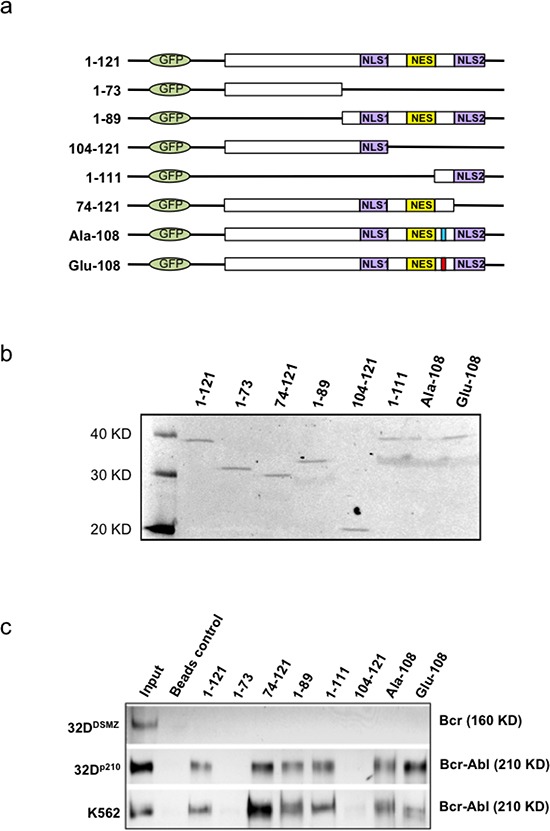
Mapping and modeling of apoptin motif responsible for its interaction with BCR-ABL1 **(a)**. Schematic representation of apoptin deletion mutants tagged with N-terminal GFP. **(b)**. Immunoblot showing the expression of deletion mutants and wild type apoptin (1–121) transfected into PC-3 cells and immunoprecipitated with anti-GFP antibody 18 h post-transfection. **(c)**. Apoptin CO-IP experiment from transfected 32D^DMSZ^, 32D^p210^ and K562 cells with various mutant forms of apoptin; Bcr-Abl was identified in the immuno-precipitates of full-length apoptin and apoptin derivatives that harbored amino acids from 74-100 (includes the proline rich region, aa: 81-86) indicating a part of this region of apoptin is important for the interaction with Bcr-Abl^p210^.

In order to identify the precise nature of apoptin and BCR-ABL1 interaction in CML cells we mapped the sites on apoptin responsible for interaction with specific region of BCR-ABL1. The murine bone marrow derived 32D^DMSZ^, 32D^p210^ cells and human CML cell line K562 were transfected with different apoptin mutant constructs by lipofectamine protocol. Schematic diagrams and the expression of these mutant derivatives of apoptin tagged with an N-terminal GFP and expressed in 32D^DMSZ^ cells are shown in figure 3a and 3b. In the experimental groups apoptin was immuno-precipitated by murine anti-GFP antibody from the lysates of transfected cells expressing various forms of mutant apoptin conjugated with GFP and the protein complexes were analyzed to detect the presence of Bcr-Abl^p210^ by immunoblotting. BCR-ABL1^p210^ was found in the immuno-precipitates of full-length apoptin and apoptin derivatives that harbored aa 74-100 (including the PRS), implying that this region of apoptin is important for interaction with BCR-ABL1^p210^ wt (Fig. 3c). Interestingly, in this model system the mutants Ala-108 and Glu-108 where the Thr-108 residue of apoptin was replaced by alanine or glutamine respectively (Ala-108 mutation of apoptin turns it resistant to phosphorylation and is non-toxic to the cells, whereas the Glu-108 mimicked the phosphorylated apoptin and retained toxicity) were also able to interact with BCR-ABL1^p210^ (Fig. 3c).

### Synthetic proline rich sequence of apoptin (aa: 81-90) induces *cell death* in Bcr-Abl1 expressing cells and is toxic to imatinib resistant patient derived primary samples

**Figure 4:**
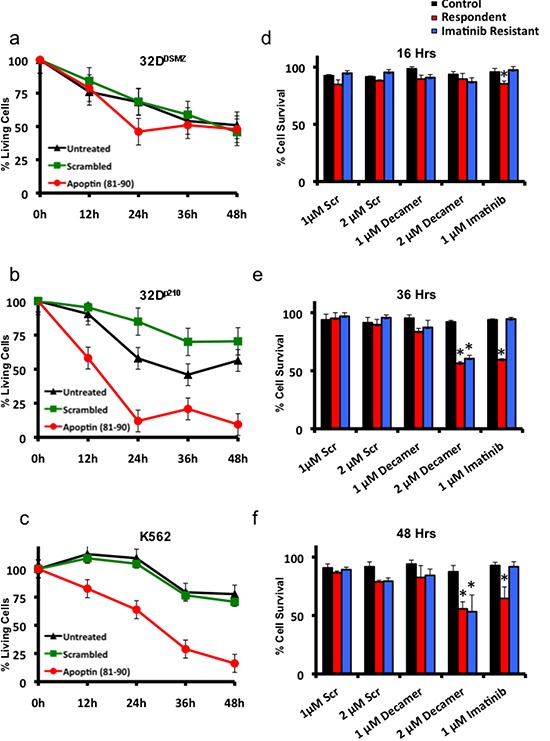
Apoptin-derived proline-rich motif preferentially kills BCR-ABL1-positive cells **(a)** The effects of Tat-conjugated apoptin derived peptide on the survival of Bcr-Abl non-expressing 32D^DSMZ^ cells (MTT assay). **(b)** the effects of Tat-conjugated apoptin derived peptide on the survival of Bcr-Abl expressing 32D^p210^ cells (MTT assay). **(c)** the effects of Tat-conjugated apoptin derived peptide on the survival of Bcr-Abl expressing K562 cells (MTT assay). **(d, e, f)**. Both Imatinib responsive and resistant patient samples are sensitive to apoptin derived decapeptide but not healthy donor samples. MTT assay results show a time dependent cell death by apoptin decapeptide at 16h, 36h and 48h. N=3. *p<0.05.

To study the biological activity of the apoptin derived cell-penetrating synthetic peptide on murine 32D^p210^ cell lines and human K562 cell lines expressing Bcr-Abl1^p210^, Tat-conjugated peptide (rkkrrqrrr-**PKPPSK**KRSC) was added at a concentration of 1μM to the growing cells in culture and cell survival was estimated by MTT cell survival assay at different time points over a period of 48 hours. The murine IL3-dependent primary hematopoietic murine cell line 32D^DSMZ^ was used as the control cell line. In another set of parallel experiments a scrambled Tat-conjugated peptide sequence (rkkrrqrrr-PRRPSRSPKC) was used as treatment control. The results obtained from these three cell lines (32D^DSMZ^ 32D^p210^ and K562) treated with both test and control peptides are portrayed in figure 4a-c respectively. Cells grown without any treatment (control) were set to 100% proliferation and the cell survival was expressed as normalized average. As shown in figure 4a apoptin derived decapeptide treatment does not show any significant cellular toxicity among 32D^DSMZ^ as compared to control and scrambled peptide treated cells. However, apoptin-derived decapeptide induced significant inhibition of cell proliferation and/or cell death among 32D^p210^ as shown in figure 4b compared to control and scrambled peptide treated counterparts. These results further confirm the anti-proliferative effect of apoptin and apoptin derived peptides mediated through their SH3 domain interacting proline rich regions. Interestingly, similar peptide treatments on the BCR-ABL1^p210^ expressing K562 cells also have similar results (Fig. 4c).

Inspired from these *in vitro* results, we further tested the anti-proliferative effect of apoptin-derived decapeptide (**PKPPSK**KRSC) on imatinib sensitive (N=3) and imatinib resistant (N=3) patient samples and compared to cells derived from healthy donors (N=3). All the experiments were conducted along with scrambled peptide (PRRPSRSPKC) as a control treatment and Imatinib as a positive control. As shown in figure 4e and 4f apoptin derived decapeptide treatment (1μM and 2μM) for 36 h and 48 h respectively showed decreased cell survival among imatinib responsive and resistant patient samples without showing any difference among healthy samples. Hence, Imatinib treatment (1μM) did not cause any significant difference in cell survival among imatinib resistant patient derived samples but did show significant decrease in cell survival among responsive samples (Fig. 4d-f). Scrambled peptide treatment at similar concentration as apoptin decapeptide (1μM and 2μM) did not show any significant difference in cell survival among the samples.

### Synthetic Apoptin decapeptide inhibits c-Myc phosphorylation and thus impedes cell proliferation of *BCR-ABL1* expressing cells

**Figure 5:**
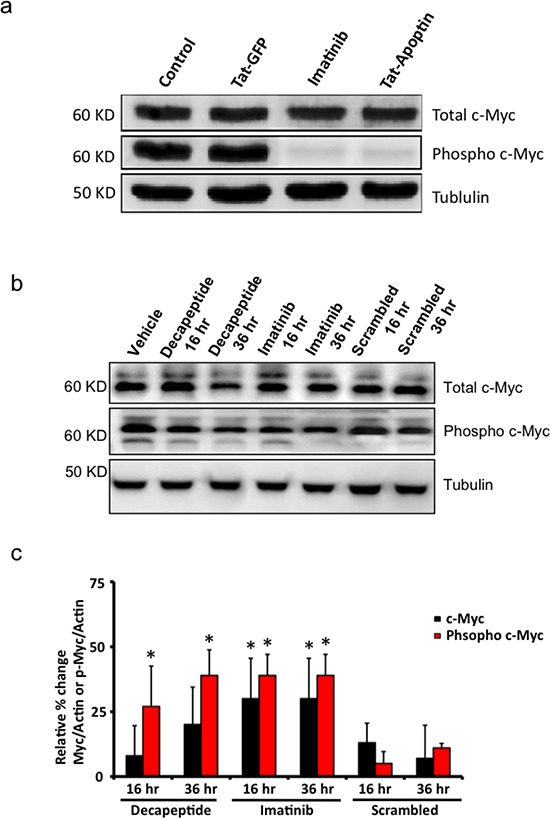
Downstream effects of Bcr-Abl inhibition by apoptin or its bioactive decapeptide (**a**) Western blot image showing inhibition of c-Myc phosphorylation by Tat-apoptin among K562 cell lines similar to that of Imatinib. (**b**) Bioactive apoptin decapeptide shows similar attenuation of phosphorylation of c-Myc by 36 h but not the scrambled peptide sequence. (**c**) Quantification of western blot data from figure 5b. N=3. *p<0.05.

In our earlier work [19], we showed that apoptin interacts directly with BCR-ABL1 and inhibits the phosphorylation of its down stream targets. Here we tested the effects of Tat-Apoptin, and our apoptin-decapeptide on the expression levels of c-Myc and its active phosphorylated form that enhances cell proliferation among BCR-ABL1 expressing K562 cell lines. Western blot image in figure 5a shows that Tat-Apoptin and Imatinib treated cells showed hampered phosphorylation of c-Myc compared to control and Tat-GFP treated cells. Similarly we tested the efficacy of synthetic apoptin-decapeptide in inhibiting c-Myc phosphorylation among K562 cells. As shown in the figure 5b apoptin decapeptide treated cells showed inhibited phosphorylation of c-Myc by 36 h similar to Imatinib treated cells at the same time period compared to control treated and scrambled peptide treated cells at the respective time interval. A quantified data of Western blot is shown in figure 5c depicting similar results.

## DISCUSSION

Previously, we showed that Tat-apoptin, a cell-penetrating conjugate of apoptin strongly binds to the SH3 domain of BCR-ABL1, modifies the phosphorylation and thus inhibit the activity of BCR-ABL1 followed with an attenuation of several of its downstream targets [19]. These changes lead to the identification of anti-proliferative effect and induction of intrinsic apoptotic pathways in the rapidly growing CML cells by apoptin [19]. Using human CML cells K562 and BCR-ABL1^p210^ expressing murine cell line 32D^p210^ as a model, which are highly responsive to apoptin, we further ventured into identifying the smallest fragment of apoptin that can inhibit BCR-ABL1 activity and thus mimic the anti-cancer activity of apoptin. In this study, employing pull-down assays we have successfully identified BCR-ABL1 interaction domain of apoptin located between amino acids 81-90. Furthermore, we show that this apoptin-derived decapeptide's anti-cancer activity favorably compares to imatinib. We have further tested the activity of apoptin decapeptide among CML cell lines K562 and 32D^p210^, and most importantly among CML-samples derived from Imatinib responsive and -resistant patients. We show that the apoptin decapeptide is toxic even for the Imatinib resistant patient samples along with the responsive samples.

In BCR-ABL1, the fusion of Bcr sequences to the Abl-SH3 domain abrogates the physiologic regulation of the tyrosine kinase Abl. This fusion protein is capable of inducing uncontrolled auto-phosphorylation that activates the downstream cell growth, proliferation and anti-apoptotic pathways. These altered signaling cascades lead to a series of alterations in the behaviors of CML cells like diminished adhesion to the stromal environment, degradation of inhibitory proteins, activation of mitogenic signaling and inhibition of apoptosis. The human (K562) and murine cell lines (32D^p210^) express BCR-ABL1 and are thus rapidly growing with a high cytoplasmic BCR-ABL1^p210^ pool. Thus the cell culture conditions of these cell lines resemble blast crisis stage of CML. Furthermore, as it is in CML, the central mitogenic Ras-MAPK cascade is also activated in these cell lines. Our findings corroborate well with previous studies involving a similar approach directed towards the Grb2-SoS-Ras-MAP kinase (Erk) pathway [21]. In those experiments Kardinal and colleagues applied small, high affinity peptides blocking the N-terminal SH3 domain of Grb2. Their results indicate that peptide based inhibitor of BCR-ABL1 kinase or its down-stream targets could be valuable anti-CML tool if combined with conventional cytotoxic therapy [21]. Taking a lead from these studies and experimental models here we studied to derive apoptin based small peptides that interact and target BCR-ABL1 mediated effects.

To test the possible interaction of apoptin with BCR-ABL1 an attempt was made to identify the interaction partners for apoptin by using SH3 specific array that revealed that SH3 regulatory subunit of c-Abl is a major candidate interacting with recombinant apoptin (Fig. 2a). The interaction of BCR-ABL1 during apoptin's treatment was confirmed by GST-apoptin pull down assay and apoptin/BCR-ABL1 co-immunoprecipitation assay in BCR-ABL1 expressing murine (32D^p210^) and human cell line (K562) transfected for the expression of GFP-Apoptin (Fig. 2b and 2c). The same set of cell lines was used for immunofluorescence studies to view protein-protein interaction directly in the cell. As seen in the transfected mouse cell systems, the transiently expressed GFP conjugated apoptin co-transport cytoplasmic BCR-ABL1 protein pool to the nucleus and induced apoptotic response [19].

We also observed that apoptin unlike imatinib was effective both against BCR-ABL1-positive and BCR-ABL1-negative but transformed cells (Fig. 1c) as apoptin has multiple and diverse targets within the cancer/transformed cells as opposed to Imatinib. Thus, hypothetically apoptin based therapeutics would be not only more effective, but they would be less prone to the development of a resistance. So, we further followed this study to elucidate the minimal region of apoptin that is capable of interacting with BCR-ABL1. Using various deletion constructs of apoptin and employing pull down assays we show that apoptin constructs processing proline rich region of apoptin ranging from amino acids 81-90 were able to pull down BCR-ABL1 (Fig. 3c). Encouraged by these results we made synthetic constructs of the apoptin decapeptide (amino acids 81-90) and showed that cell permeable Tat-conjugate of this peptide is toxic among BCR-ABL1 expressing murine 32D^p210^ and human cell line K562 (Fig. 4b and 4c) but not toxic to 32D^DSMZ^ cells (Fig. 4a). Further, *ex vivo* cellular toxicity of apoptin decapeptide among both Imatinib resistant and Imatinib susceptible CML patient derived samples (Fig. 4 e-f) and mimicking the full length apoptin in inhibiting the BCR-ABL1 downstream signaling of c-Myc (Fig 5 a-c) illustrates the potentiality of the apoptin derived peptide for further treatment of CML.

**Figure 6:**
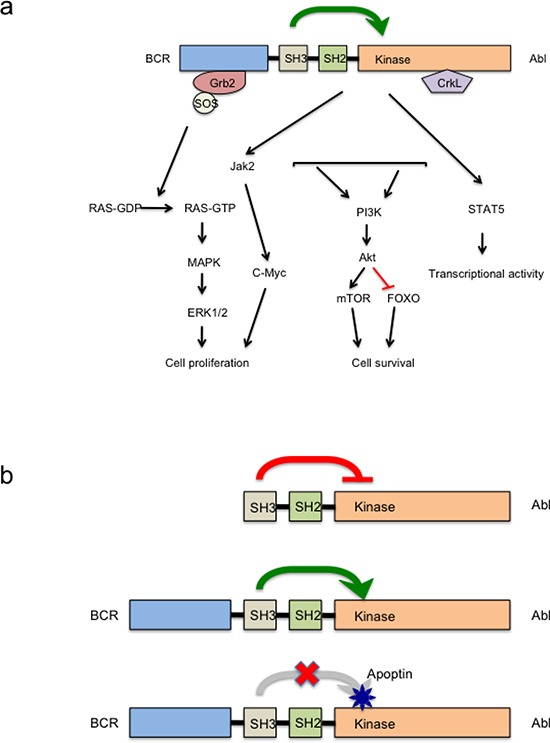
Anticancer action of apoptin targeting Abl/Bcr-Abl pathway **(a)** Major signaling pathways activated in Bcr-Abl transformed cells. **(b)** Schematic diagram of apoptin induced Bcr-Abl kinase inhibition. UPPER PANEL- In normal c-Abl the SH3 domain acts as an endogenous inhibitor of its own kinase (SH1 domain) due to its interaction to a proline rich sequence of the same molecule. MIDDLE PANEL: In the fusion protein Bcr-Abl, the attachment of Bcr to the SH3 domain of Abl disrupts the attachment to this internal proline rich sequence and abrogates the kinase inhibition leading to autophosphorylation and transactivation of other oncogenic kinase pathways. LOWER PANEL: Strong interaction between the proline rich sequence of apoptin and the SH3 domain of oncoprotein Bcr-Abl leads of reinstitution of this inhibition on Bcr-Abl kinase.

Along with our previous study [19] and in this study we verified the specific interaction domains of apoptin and BCR-ABL1^p210^ fusion protein. As demonstrated, apoptin works by binding to the SH3 domain of BCR-ABL1 and possibly acting as an adaptor molecule it prevents the activation of the kinase domain (SH1) [19]. Thus, the ATP binding and phosphorylation of BCR-ABL1 is downregulated, hence loss of signal leads to down regulation of cell proliferation and activation of intrinsic apoptotic pathways. The hypothesis clarifying the mechanism of apoptin induced down regulation of activated BCR-ABL1 kinase in CML is summarized in figure 6b.

In summary, the present study establishes the minimal region of apoptin interaction domain with BCR-ABL1 and further shows the anti-cancer effect of the cell-permeable version of apoptin decapeptide (aa 81-90) on the BCR-ABL1 expressing mouse and human cell lines. The synthetic apoptin decapeptide with a proline rich region is able to inhibit cell proliferation of both imatinib-resistant and imatinib sensitive patient samples. We further show that the apoptin synthetic peptide is able to inhibit BCR-ABL1 down stream target c-Myc with a comparable efficacy to full-length apoptin and Imatinib. Thus, for the first time, we show the therapeutic potential of apoptin decapeptide for treatment of CML. Furthermore, the apoptin decapeptide may represent a solution to three major challenges in the current therapy of imatinib-resistant CML: (*i*) inspite the good prognosis for many CML patients on imatinib, 20-30% of them develop resistance to imatinib [3, 22] and 7-9% of patients progress to accelerated phase or blast crisis on imatinib treatment [7, 8, 23]. Thus, the new apoptin-based targeted therapy alone or in combination with Imatinib could prevent the resistance development and progress to accelerated phase or blast crisis. (*ii*) The second advantage of new apoptin-based mono or combination therapy could be to obtain a long-term cure for CML treatment by inducing deeper and faster molecular and cytogenetic responses and successful discontinuation of therapy [24] without experiencing an early molecular relapse. (*iii*) Finally, 10% of patients discontinue TKI therapy due to concentration-dependent and -independent adverse events [25]. The employment of apoptin decapeptide-based combination therapy may lower concentration dependent adverse events because one could diminish the TKI doses.

## MATERIALS AND METHODS

### Cell lines, plasmids, Cell death and cell proliferation assays, antibodies and reagents

All cell culture media and supplements were from Gibco BRL. 32D^DSMZ^ and 32D^p210^wt:b3:a2/e13:a (described as 32D^p210^) [26], a BCR-ABL1 variant, were grown in RPMI-1640 medium supplemented with 20% FBS (Hyclone), 100μg/ml penicillin and 0.1 μg/ml streptomycin (Gibco BRL), 10mM HEPES, 2mM L-Glutamine, 0.13mM L-Asparagin, 0.05 nM 2-Mercaptoethanol, 1mM Na-Pyruvate, and non-essential amino acids. 32D^DSMZ^ cells are strictly murine IL3-dependent, so the media was supplemented with 10 % media supernatant from WEHI-3B cells [26]. The human CML cell line K562 (ATCC^®^ # CCL-243™) was cultured in ATCC recommended Iscove's modified Dulbecco's medium with 4mM L-glutamine adjusted to contain 1.5g/L sodium bicarbonate, 10% FCS (v/v) and antibiotics [27, 28]. All cells were grown at 37°C with 5% CO_2_ in a humidified incubator and maintained in a logarithmic growth phase.

The following plasmids were used: GFP, GFP-Apoptin (apoptin cloned into pEGFP-C1 vector, Clontech), GST, GST-Apoptin (apoptin cloned into PGEX-2T vector, Amersham Biosciences), apoptin mutants were previously described [29].

Chemicals and antibodies were purchased from Sigma-Aldrich^®^ Inc. (Sigma, Oakville, ON, Canada) Abcam^®^ Inc. (Cambridge, MA, USA) or Cell Signaling Technology, Inc. (Danvers, MA, USA). The following antibodies were used: monoclonal murine/rabbit anti- Bcr-Abl/anti-Bcr (Abcam Inc. UK), murine anti-Akt, rabbit anti-c-Myc, rabbit anti-Phospho c-Myc and the murine monoclonal anti-apoptin antibody (kind gift from Dr. D. Jans, Australia).

### CML Patient derived Primary cells

Peripheral blood samples were obtained from 6 CML-patients positive for BCR-Abl1 (3 patients showing positive response to Imatinib treatment (Imatinib responsive) and 3 patients that did not respond to imatinib therapy (Imatinib resistant) and 3 healthy adult donors from King George's Medical University, erstwhile Chhatrapati Shahu Ji Maharaj Medical University, Lucknow, Uttar Pradesh, India, after informed consent according to the approved institutional ethical guidelines. Mononuclear cells were isolated by Histopaque gradient centrifugation (density 1.077 g/mL; Sigma-Aldrich). After washing with PBS cells were suspended in RPMI-1640 supplemented with 10% FBS and were immediately used for experiments.

### TransSignal SH3™ Domain Array

The SH3 domain array was performed according to the manufacturer's protocol (Panomics, Inc. Redwood City, CA, USA). In brief, the TransSignal SH3™ Domain Array1 membrane was incubated with purified Tat-Apoptin to allow protein-protein interaction, and after necessary washing steps an image of these interactions was acquired on high performance chemiluminescence film (Hyperfilm™ECL, Amersham Biosciences). The proteins in the array are spotted in duplicates. Histidine tagged ligands have been spotted along the bottom and in duplicate along the right side of the membrane for alignment purpose.

### Transfection of mammalian cells

Different mammalian primary and cancer cells were transfected with the desired plasmids by Lipofectamine^TM^2000 (Invirogen® Canada Inc. Burlington, Ontario, L7P 1A1) reagent. The cells were plated in an antibiotic free medium 24 h prior to transfection and plasmid DNA and transfection reagent were prepared as per manufacturers recommendations (1:2.5 ratio of DNA to reagent). Following the incubation, the DNA-lipid mixture was gently added directly to the cells that had previously been rinsed with PBS and replaced with fresh medium.

### GST-pull down assay and protein identification, co-immunoprecipitation (Co-IP), Western blotting

The GST and the recombinant GST-apoptin proteins were purified according to the manufacturer's protocol using glutathione sepharose beads (Amersham Biosciences^®^). GST-pull down assay was performed to detect the interacting partners of apoptin. Briefly, either purified GST or GST-apoptin along with total 32D^p210^ or K562 cell lysate were immobilized on glutathione sepharose beads overnight at 4°C in IP buffer with protease and phosphatase inhibitors (50mM Tris-HCl pH 8.0, NaCl 150mM, ND-40 0.5%, EDTA 1mM, PMSF 1mM, NaF 10mM, Na_3_VO_4_ 1mM, β-glycerophosphate 25mM). Beads were washed at least six times with ice-cold lyses buffer and the bound proteins were detected by Western blotting.

For Co-IP experiments, 2-5 μg of antibody was added to 100-500 μg of cell lysate and incubated (4h at 4°C) and then 100 μl of equilibrated 50% protein-G Sepharose beads (Amersham Pharmacia Biotech^®^) were added to the protein-antibody immune complexes and incubated (1h at 4°C). Beads were washed (6x) with lyses buffer and after the final wash, beads were suspended in SDS sample buffer and resolved on SDS-PAGE gel, and the immunoprecipitated protein was detected by Western blotting as previously reported [30-32]. Briefly, about 30μg protein lysates were resolved by SDS PAGE, transferred to PVDF-membrane (Amersham Biosciences®) followed by membrane blocking with 5% non-fat dry milk powder/tris-buffered saline with 0.25% v/v Tween-20 5% BSA. Membranes were washed and incubated with an appropriate secondary antibody conjugated with horseradish peroxidase (HRP) for 45min at room temp and detected by using enhanced chemiluminescent (ECL) staining (Amersham Biosciences).

### Immunocytochemistry and fluorescent imaging

Cells were allowed to attach overnight, transfected with appropriate plasmids and after 16-18h of incubation cells were collected, washed with PBS, and fixed in 4% w/v paraformaldehyde /PBS. Thin smear of cells was prepared on standard microscope glass slides and air-dried. Cells were permeabilized (0.1% triton X-100/PBS), blocked (5% BSA/PBS, 1h) and incubated overnight at 4°C with an appropriate primary antibody, followed by incubation with Cy3 or FITC conjugated secondary antibody. Slides were mounted with Vectashield® containing DAPI. Fluorescence signals were acquired using Zeiss fluorescent microscope and analyzed by Zeiss Axiovision 3.1 software.

### Tat-mediated protein transduction

The recombinant Tat-GFP and Tat-Apoptin proteins were expressed in BL21 bacterial cells and purified as described before [33]. Cells were seeded in either 6 well or 12 well culture plates (Corning Incorporated COSTER). Tat-fusion proteins were diluted in culture medium to the desired concentrations (1mM) and added directly to the cells. Cells were incubated with Tat-fusion proteins at 37°C in a 5% CO_2_ humidified incubator for 16h.

### Synthesis of short decapeptides

Apoptin derived decapeptide (PKPPSKKRSC) and scrambled peptide (PKKPSKKRSC) was synthesized on symphony automated peptide synthesizer (Protein Technologies Inc., Tucson, AZ, USA) using standard fluorenylmethoxycarbonyl (Fmoc) chemistry with HCTU (ChemPep Inc., Wellington, FL, USA) as the activating reagent. The synthesis was performed on a 0.05 mmol scale with Fmoc-Cys (Trt)-PEG-PS resin (Applied Biosystems, Sweden) using a five-fold excess of amino acid in each coupling. The peptides were cleaved from the resin by treatment with a mixture of trifluoroacetic acid (TFA), and water (95:5 v/v; 10 mL per gram of polymer) for 1h at room temperature. After filtration, TFA was evaporated and the peptides were precipitated by the addition of cold diethyl ether, centrifuged and lyophilized. The crude products were purified by reversed-phase HPLC on a semi-preparative C-18 column (Grace Vydac) and identified from MALDI-TOF spectra (Applied Biosystems Voyager DE-STR, Stockholm, Sweden).

### Cell death and cell proliferation assays

Cell proliferation was assessed by MTT assay, whereas cell death was measured by propidium iodide uptake. Both assays were performed as previously described [34, 35].

### Statistical Analysis

Unless stated otherwise, all normalized band intensity data were statistically analyzed by *student's t test assuming equal variance* using Microsoft® Excel software. The variance patterns in each set of data were previously checked by ANOVA from Excel data analysis tool package and Prism (version 6.0b) software. Error bars in all the graphs represent standard deviation.

## Abbreviations

ALL: acute lymphoblastic leukemia
CML: chronic myeloid leukemia
CO-IP: Co-immunoprecipitation
Cy3: Cyanine3
DAPI: 4′,6-diamidino-2-phenylindole
FITC: Fluorescein isothiocyanate
HRP: horse radish peroxidase
MAPK: Mitogen activated protein kinase
MTT: 4,5-Dimethylthiazol-2,5-diphenyltetrazolium bromide
nr-TK: non-receptor tyrosine kinase
PI3K: Phosphoinositide inositol 3 kinase
PI: Propidium iodide
r-TK: receptor tyrosine kinase
SH2: Src homology 2 domain
SH3: Src homology 3 domain
STI: signal transduction inhibitor
TK: tyrosine kinase
